# Effectiveness of the Health Extension for Diabetes Program, a DSMS Program Delivered In-Person and Online: A Quasiexperimental Comparative Study

**DOI:** 10.1155/jdr/8092802

**Published:** 2025-10-06

**Authors:** Michelle A. Parisi, Danielle McFall, Windsor Westbrook Sherrill, Michelle Stancil, Christina J. Dietz, Samantha Kanny, Maria M. Rossi

**Affiliations:** ^1^Nutritional Sciences Department, University of Georgia, Athens, Georgia, USA; ^2^Cooperative Extension Services, Clemson University, Clemson, South Carolina, USA; ^3^Department of Public Health Sciences, Clemson University, Clemson, South Carolina, USA; ^4^Prisma Health-Upstate, Diabetes Care and Education, Greenville, South Carolina, USA; ^5^Department on Internal Medicine, The Ohio State University Wexner Medical Center, Columbus, Ohio, USA; ^6^School of Health Research (CUSHR), Clemson University, Clemson, South Carolina, USA

**Keywords:** Cooperative Extension, diabetes self-management, health education, Health Extension for Diabetes, online learning

## Abstract

**Background:**

Diabetes is a complex, prevalent condition. Effective diabetes self-management requires knowledge, skills, and strategies to prevent or delay the onset of diabetes-related complications. This study evaluated the effectiveness of delivery methods, online versus in-person, for the 4-month-long Health Extension for Diabetes (HED) program, a self-management support program.

**Methodology:**

A quasiexperimental comparative study design assessed whether online HED delivery was as effective as in-person delivery and whether group differences affected health outcomes. Multivariate analysis of covariance (MANCOVA) was conducted to assess changes in participants' weight, BMI, HbA1c, diabetes self-efficacy, knowledge, and self-management behaviors. This study included a total of 1018 participants (*o*nline = 613; *i*n‐*p*erson = 405). The online program was delivered synchronously via video or phone conferencing platforms, while in-person sessions occurred at a community location. Survey and biometric data were collected at baseline and upon program completion to evaluate pre- to postprogram changes.

**Results:**

Online HED participants were mostly White non-Hispanic, significantly younger, more educated, and had higher income than in-person participants. Regardless of the delivery modality, positive changes pre- to postprogram were seen across all biometric and diabetes outcome measures. Significant differences between delivery modalities were observed for diabetes knowledge, with individuals in online groups demonstrating significantly higher knowledge scores at both assessment points. For weight and BMI, both modalities showed significant improvements.

**Conclusion:**

Online and in-person HED modalities effectively improved diabetes self-management, reaching two different demographic groups. Demographic differences between groups underscore the importance of offering multiple participation modalities to ensure accessibility and engagement across diverse populations. The HED program model, using a community-clinical linkage through Cooperative Extension, is an effective model for improving health outcomes of participants enrolled in both online and in-person DSMS programs. Future research should focus on how different participant characteristics influence engagement and long-term diabetes outcomes across delivery modalities.

## 1. Introduction

Diabetes is a complex, chronic disease that affects 11.6% of the population (38.4 million people) [1] in the United States. The financial impact of diabetes in the United States is extensive and has increased over time, reaching nearly $413 billion in 2022 [1]. Expenses related to diabetes come from both direct medical expenses, such as diagnosis, medications, and treatment of complications, and indirect costs, including loss of productivity, early retirement, and disability [2]. The complications of diabetes account for 50% of direct costs and include comorbidities like retinopathy, neuropathy, heart disease, and kidney disease [1, 3]. Strategies to prevent or manage complications associated with diabetes are critical for reducing comorbidities and costs associated with this disease.

Diabetes self-management education and support (DSMES) is a well-established strategy that can help individuals with diabetes decrease their risk for diabetes complications. Fitzpatrick et al. [4] conducted a randomized trial to compare a formalized DSMES program to usual patient care. Researchers found that participants receiving DSMES intervention had substantial improvements in behavioral outcomes and clinical outcomes compared to usual care. In 2020, a consensus report from the American Diabetes Association (ADA) [5] provided evidence-based recommendations for the care of patients with diabetes. Authors provided evidence for the effectiveness of DSMES programs on improved glycemic control [6], lower risk of complications [7], and improved psychosocial parameters like quality of life, knowledge, and self-care behaviors [8]. More recently, Ernawati et al. [9] conducted a systematic review of the literature and confirmed that DSMES programs are an effective method for improving self-management behaviors and clinical outcomes for people with diabetes.

DSMES programs have been delivered using a variety of methods. Alonso-Carril et al. [10] compared the effectiveness of online DSMES and in-person DSMES programs through a systematic review of the literature. Researchers found that online DSMES was comparable to in-person interventions in improving hemoglobin (HbA1c) levels, specifically in people with Type 1 diabetes (T1D). Interventions that were focused on weight management strategies for people with Type 2 diabetes, however, were more effective at reducing HbA1c when delivered online than in-person interventions.

Molavynejad et al. [11] conducted a three-arm, randomized controlled trial to compare educational interventions delivered online and in-person on biometric measures for people with Type 2 diabetes. A total of 378 patients with Type 2 diabetes mellitus (T2DM) were randomly assigned to online education, in-person education, or control groups. Educational content was similar between the online education and the in-person education, focusing on nutrition, healthy cooking, and glucose self-monitoring. However, delivery of the content online was provided through recorded videos versus synchronous educational sessions. HbA1c and lipid levels were collected before participation and again at 3 months postparticipation. Results showed significant improvements in weight loss, HbA1c, and most lipid levels from pre- to postparticipation in both online and in-person interventions (mean changes of 91% and 84%, respectively, *p* = 0.001). The authors concluded that both online education and in-person education produce positive results for people with Type 2 diabetes.

Health Extension for Diabetes (HED) is a community-based diabetes self-management support program designed to complement DSMES services. The ADA designated HED as a “practice-tested support program” based on documented results of program effectiveness [12]. HED was developed in 2017 by researchers at a South Carolina (SC) land-grant university in partnership with DSMES clinicians from a large, regional healthcare system. The program was designed to be delivered using traditional in-person facilitation methods through the Cooperative Extension System with “extension agents” serving as health paraprofessionals in community settings [13]. Extension agents complete extensive training in diabetes education methods, program facilitation techniques, and resource navigation for social determinants of health (SDOH). CDCES specialists support the extension agents for participant questions and topics deemed “out-of-scope” for health paraprofessionals.

A pilot study of the HED program was completed in a sample of 149 individuals. Participation in the HED program resulted in significant improvements in diabetes self-care activities from pre- to postprogram participation [13, 14]. Researchers found that activities related to self-management of participants' average diet, specific diet, and blood glucose testing increased significantly by almost 1 day/week (+0.85, +0.74, and +0.60 days, respectively; respective *p* values < 0.05). Other self-management activities that included exercise and foot care increased significantly by more than 1 day/week (+1.01 and +1.22 days, respectively; *p* < 0.001). In 2020, HED was adapted for delivery through an online platform, and HED became available in both online and in-person settings. Therefore, we conducted a quasiexperimental study to compare the effectiveness of the online HED program to that of the traditional in-person HED program.

## 2. Methodology

A quasiexperimental comparative study design was used to evaluate and compare the effectiveness of the HED program between individuals receiving HED through online cohorts and individuals receiving HED through in-person cohorts. Within-arm analysis was completed to evaluate pre- to postprogram self-efficacy, diabetes care knowledge, diabetes self-management activities, and self-reported weight, BMI, and HbA1c. Between-arm analysis was used to determine if the interventional delivery method produced inferior or superior results over the control method. In-person cohorts served as this study's standard delivery method because all cohorts were originally delivered in-person. Also, the ADA practice-tested designation was obtained based on in-person program delivery, and the in-person cohorts represented the established standard practice of program delivery through Cooperative Extension.

### 2.1. HED Curriculum

The “ADCES7 Self-Care Behaviors” framework [15] and the “ADA: Life With Diabetes Teaching Outlines” [16] provide the basis for the HED curriculum. The ADCES7 Self-Care Behaviors is a proven framework for diabetes self-management [15]. It is structured around target behaviors identified by the ADCES required to facilitate positive diabetes-related outcomes and a decreased risk of complications [15].

The HED curriculum is composed of eight structured education sessions that are delivered every other week in small group cohorts [13, 14]. The education sessions include topics such as healthy eating, physical activity, problem-solving, healthy coping, and medications and monitoring. Extension agents facilitate seven of the eight group education sessions, and a CDCES partner delivers one session on medications, monitoring, and complications of diabetes. Each structured education session provides approximately 1 h of interaction with participants. In the weeks between the structured education sessions, follow-up calls are provided to assist participants with health and SDOH-related behavior change challenges. The curriculum is reviewed and updated annually by the program coordinator in conjunction with a CDCES based on the ADA Standards of Care in Diabetes [15].

#### 2.1.1. Standard Delivery Method: HED In-Person Program

Participants of each cohort were required to complete at least six of the eight 1-h education sessions to graduate from the program. The in-person HED program served as the standard by which online programs were compared. Program delivery followed the processes described by Dietz et al. in 2022 [13]. In-person facilitation was delivered in community-based locations. The 1-h structured education sessions were delivered every other week in a secure, private room. A partnering CDCES joined either in-person or online to facilitate the structured education session about medication and glucose monitoring. All extension agent facilitators maintained a defined scope-of-practice for nonlicensed health professionals [17]. The CDCES–extension agent partnership formalizes a process that allows participants to ask questions deemed “out-of-scope-of-practice” for the extension agent. Cohort delivery dates are established and scheduled by the extension agent and shared with the CDCES. Throughout the cohort, if out-of-scope-of-practice issues arise during sessions, the extension agent contacts the CDCES, who responds accordingly. If questions are highly individualized, a referral is made for more formal education with the CDCES outside of the program delivery. Additionally, participants received weekly follow-up using their preferred communication mode, including phone calls, emails, or text messages. Follow-up interactions were used to discuss progress toward personal goals, assist with clinical and social resource navigation, and address barriers to participation and self-care behavior change. Participants were strongly encouraged to join online support groups delivered through the health system in addition to the HED sessions. The total length of the in-person program was approximately 4 months.

#### 2.1.2. Adapted Delivery Method: HED Online Program

The HED program was delivered in small groups (cohorts) synchronously through an online video conferencing platform. To minimize technology barriers, facilitators assisted participants individually to ensure they were able to access the initial session. At the first session, time was allotted for a short orientation of the platform and skills needed for full participation. Participants who did not have access to a computer or the internet but wanted to participate virtually were given a corresponding telephone number to engage through telephone conferencing. All program handouts and materials corresponding to the upcoming session were emailed or mailed to participants before each session.

Similarly to in-person delivery, participants were required to complete at least six of the eight structured, 1-h education sessions to graduate from the program. They were also strongly encouraged to participate in online support sessions offered in the weeks between core sessions. Similar to online delivery, all extension agent facilitators maintained a defined scope-of-practice for nonlicensed health professionals [17]. A medications and glucose monitoring session was delivered by a partnering CDCES who joined the group through videoconferencing. Throughout each cohort of the program, the CDCES answered questions deemed out-of-scope-of-practice for extension agents through a previously agreed-upon chain of communication. Each participant also received weekly follow-up in the form of either a phone call, email, or text message, based on their personal preference. Follow-up interactions were used to discuss progress toward personal goals, assist with clinical and social resource navigation, and address barriers to participation and self-care behavior change. The total length of each cohort of the program was approximately 4 months.

### 2.2. Sampling

Recruitment strategies for both the intervention and control programs included promotional materials published on social media, Eventbrite, university calendars, websites, and electronic patient health records (i.e., MyChart messaging and SmartPhrases). Local health fairs were also used to promote both program modalities in the region. Direct HED program referrals from healthcare providers were channeled through a HIPAA-approved, centralized communication hub at the university. The in-person program employed additional strategies for recruitment that involved the distribution of hardcopy promotional flyers to local businesses, healthcare provider offices, schools, churches, and community bulletin boards. All recruitment materials included a QR code and URL link that were associated with an “interest survey.” Individuals interested in participating in the HED program were encouraged to go to the web address and complete the survey that collected names, contact information, eligibility information, and county of residence. A member of the research team then contacted the interested individual, verified eligibility, and if meeting inclusion criteria, was provided options for participation. Of note, in-person programs were not available in all counties in the state. If individuals did not live in a county where an in-person program was being offered, they were encouraged to participate in an online cohort. Those who lived in a county where the program was being delivered in-person were given the day, time, and location where the program was offered and then asked whether they wanted to participate in the traditional, in-person program or in a synchronously delivered online program.

### 2.3. Eligibility Criteria for the HED Program Participation

The eligibility criteria for HED are the same for both the online and in-person programs.

#### 2.3.1. Eligible Criteria

Criteria for inclusion were age (adults 18 years or older) and diagnosis of T1D or Type 2 diabetes. The decision to include individuals with both T1D and Type 2 diabetes was made under the assumption that many lifestyle behaviors for blood glucose management and prevention of diabetes-related complications are similar for both T1D and Type 2 diabetes. Individuals with a written or self-reported diabetes diagnosis, a written lab report or self-reported HbA1c value greater than 6.5%, a score of 5 or higher on the ADA Type 2 Diabetes Risk Assessment, or a practitioner referral were considered eligible to participate in the program [17].

#### 2.3.2. Exclusion Criteria

Pregnant women, individuals under 18 years of age, and individuals with end-stage renal disease (ESRD) were excluded from the study. Individuals deemed ineligible were referred to formal DSMES services, diabetes prevention programs (i.e., Diabetes Prevention Program), or other clinical or community resources deemed appropriate.

#### 2.3.3. Research Ethics Review

The Institutional Review Boards of Clemson University and Prisma Health-Upstate approved this study (Study ID: Pro00073892). Data from the ongoing longitudinal study of HED programs delivered between October 2017 and February 2025 in SC were analyzed. In both online and in-person HED programs, verbal informed consent for participation was obtained. Participant data were collected via telephone or in-person. Predata collection or enrollment was completed up to 1 month prior to the first session. Postdata collection was completed within 1 month of the final session delivery. Data collection was completed by members of the research team at the university. Self-reported body weight and most recent HbA1c values were collected before and after program participation.

### 2.4. Instrumentation

The data collection instrument tool was divided into four sections: (1) Summary of Diabetes Self-Care Activities, (2) Self-Efficacy for Diabetes (SED), (3) a modified version of the Diabetes Knowledge Questionnaire (DKQ-24), and (4) demographic characteristics that were collected. The data collection instrument was administered by a member of the research team who read the questions and possible answers to the participant and cued the participant to provide a verbal response. Individuals did not self-administer the data collection surveys.

#### 2.4.1. Summary of Diabetes Self-Care Activities

The Summary of Diabetes Self-Care Activities was used to assess individual self-care behaviors surrounding diet, exercise, blood glucose monitoring, and foot care [18]. This instrument assesses the average number of days per week (on a scale of 0–7) an individual performs recommended diabetes self-management behaviors. SDSCA uses 10 questions to determine general diet, specific diet, exercise, blood glucose monitoring, and foot care, with two questions per category. The score for each subscale is the average for the associated questions [18].

#### 2.4.2. SED

The SED is an eight-item instrument created by the Self-Management Resource Center [19, 20]. This survey seeks to understand an individual's confidence in various areas of diabetes self-management. SED utilizes a 10-point scale, ranging from 1 to 10, where a higher score correlates with higher self-efficacy [21, 22]. The instrument has demonstrated test–retest reliability [19, 20, 22]. To assess the internal reliability of the instrument, Cronbach's alpha was calculated at 0.84, showing good internal reliability in our program samples [13, 18, 23].

#### 2.4.3. DKQ-24

We used the modified version of the DKQ-24.

The DKQ-24 is a 24-item version of the original 60-item Diabetes Knowledge Questionnaire, designed to assess general diabetes knowledge. Each item was scored as *correct* (1) and *incorrect/do not know* (0), with total scores ranging from 0 to 24, where higher scores indicate greater diabetes knowledge. The DKQ-24 was originally developed and validated in both English and Spanish (0.78) [24] and has since demonstrated good reliability and validity across diverse populations and languages.

#### 2.4.4. Demographic Characteristics

Demographic information included age, race, sex, ethnicity, educational attainment, and annual income.

### 2.5. Data Collection and Management

Demographic information, SED, and SDSCA were completed directly in REDCap (Research Electronic Data Capture). REDCap is a HIPAA-compliant, secure, web-based software designed to support data for research studies [25, 26]. Upon completion, study team members scored SED and SDSCA using the methodology supplied by the authors. The scores of the resulting instruments were input into REDCap.

### 2.6. Data Cleaning

**Figure 1:**
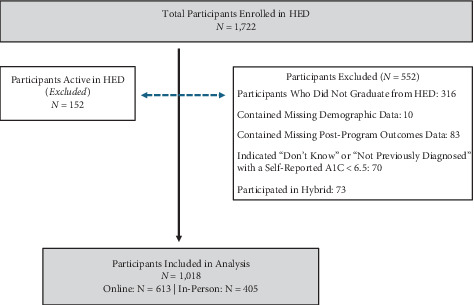
Inclusion and exclusion criteria to select participants.

For more information, see Figure 1.

#### 2.6.1. Inclusion Criteria for Data Analysis

Participants were included in the analysis if they (1) graduated from the program, (2) completed at least partial pre- and postprogram assessments, and (3) self-reported a diagnosis of diabetes (Type 1 or Type 2).

#### 2.6.2. Exclusion Criteria for Data Analysis

Participants were excluded if they reported “do not know” as their diabetes status and had an HbA1*c* < 6.5% or reported “not previously diagnosed” with no supporting HbA1*c* ≥ 6.5%. Participants who had missing demographic data were excluded to allow for consistent adjustment for possible demographic covariates in the analysis.

### 2.7. Statistical Methods

Statistical analyses were conducted using SPSS Version 28 for Windows (IBM Corp Released 2021). Descriptive statistics were computed to summarize the characteristics of the sampled population. A chi-square test of independence assessed whether categorical demographic variables, such as sex, race, ethnicity, education level, and annual income, differed between online and in-person groups. An independent samples *t*-test was conducted to compare the mean age between the intervention and standard delivery groups.

HED outcome measures were analyzed using a series of 2 (group : online and in‐person) × 2 (time : preprogram and postprogram) multivariate analyses of covariance (MANCOVAs). Time was treated as a within-subjects factor, and group was treated as a between-subjects factor, allowing us to examine within-participant changes over time while adjusting for relevant covariates and testing for group × time interactions. This approach enables simultaneous analysis of multiple outcome variables, improves interpretability, and preserves statistical power by controlling for baseline differences.

Given the structure of the outcome data, four separate repeated-measures models were conducted to assess pre- to postprogram changes across outcome domains: (1) clinical outcomes (weight and BMI), (2) clinical outcome (HbA1c), (3) psychosocial outcomes (diabetes knowledge and self-efficacy), and (4) self-care behaviors (summary scores for diet, exercise, blood glucose monitoring, and foot care). Clinical outcomes were split into two models due to differential missingness across variables. While weight and BMI were available for most participants (*N* = 978), HbA1c was self-reported and often missing (*N* = 413), as many participants were unaware of their most recent value. To maximize sample size, weight and BMI were analyzed together in a repeated measures MANCOVA, and HbA1c was analyzed independently using a repeated measures ANCOVA. Psychosocial outcomes and self-care behaviors were each analyzed using separate repeated measures MANCOVAs. Age, race, ethnicity, education level, and annual income differ significantly between groups and were included as covariates in each of the MANCOVA models.

When multivariate group or interaction effects were significant, follow-up univariate ANOVAs were conducted. For significant univariate interactions, pairwise comparisons were conducted to identify the source of the interactions by means of independent samples *t*-tests with Bonferroni's corrections.

An a priori power analysis indicated a total sample size of 121 participants was required to adequately power the MANCOVA (Cohen's *f*^2^ = 0.4) [27]. For all significant effects, partial eta squared was calculated to estimate effect size (*np*^2^ 0.01 small; 0.06 medium; 0.14 large) [28]. The alpha level was set at 0.05 for all analyses.

## 3. Results

Between 2018 and 2025, a total of 1722 participants were enrolled in 155 cohorts with a mean cohort size of 9.5 individuals (nine to 10 individuals/cohort). Over the course of 7 years of data collection, 316 individuals were lost to attrition. No differences were found between retention rates of online participants compared to in-person participation (75.92% and 79.60% retained, respectively; *p* = 0.201). A final sample size of 1018 participants completed either the online or in-person HED programs and met the inclusion/exclusion criteria for inclusion in the analysis. Participants were allowed to select their preferred modality of participation, resulting in the sample sizes *N* = 613 and *N* = 405 for the online and in-person programs, respectively. Mean retention rates for individuals continuing after two education sessions and graduating after completing at least six structured education sessions were 78.7% retention and 83.7% retention for the online group and in-person group, respectively (Table 1).

Participants in the online program differed from the in-person program participants in all demographic variables at a significance level of *p* < 0.001 (age, race, ethnicity, education level, and annual income) except sex (*p* = 0.389) (Table 2). Females made up 71.6% of participants in the online programs and 74.1% in the in-person programs. The mean age of online participants was 62.4 years, compared to 69.3 years for in-person programs. Most online participants (64.8%) self-identified as White non-Hispanics, 23.47% as Black/African Americans, and 10.9% as Hispanics. For the in-person participants, just over one-half of participants (50.6%) self-identified as Black/African American, 45.67% as White non-Hispanics, and 2.2% as Hispanics. Nearly two-thirds of the online participants, 61.4%, had an associate/technical degree or higher compared to 43.2% of in-person participants. Income levels in 29.5% of online participants were ≥$50,000 compared to 16.6% of those participating in the in-person program. Table 2 presents the demographic characteristics of our sampled population.

### 3.1. Health Outcomes Measured

To assess the impact of covariate adjustment, parallel MANCOVA models were run with and without demographic covariates. While the overall pattern of group differences remained similar, some differences in statistical significance emerged. Notably, self-care behaviors and self-efficacy, which were significant in the unadjusted model, were no longer significant after adjusting for covariates.

Results for the adjusted MANCOVA and ANCOVA analyses are presented in Table 3. For the MANCOVA models, a significant group × time interaction for clinical outcomes (*p* = 0.007), specifically for both weight (*p* = 0.002) and BMI (*p* = 0.002), indicates differential changes over time between groups. No other group × time interactions were observed for psychosocial outcomes or self-care behaviors. A significant group main effect was found in the psychosocial outcomes model (*p* = 0.009), driven by a significant difference in diabetes knowledge (*p* = 0.003). No significant group differences were observed for diabetes self-efficacy (*p* = 0.392), and no additional group main effects were identified for clinical outcomes or self-care behaviors. In the ANCOVA model, analyzing HbA1c, neither group × time interactions (*p* = 0.482) nor group main effects (*p* = 0.984) reached statistical significance.

### 3.2. Group × Time Interaction Effects

Table 4 presents pre- to postprogram changes for outcomes for which significant group × time interactions were observed. A repeated measures MANCOVA revealed a significant group × time interaction for clinical outcomes (*p* = 0.007), indicating a possible differential change in weight and BMI across delivery formats (online vs. in-person). Pairwise comparisons showed a significant decrease in weight from M = 211.6 to M = 206.9 pounds, with an average loss of 4.7 pounds (*p* < 0.001). BMI decreased from M = 34.4 to *M* = 33.6 kg/m^2^, a reduction of 0.8 kg/m^2^ (*p* = 0.001). In-person participants reported a weight reduction from M = 208.4 to M = 206.3 pounds, an average loss of 2.1 pounds (*p* = 0.002). BMI decreased from M = 33.9 to *M* = 33.6 kg/m^2^, a reduction of 0.3 kg/m^2^ (*p* = 0.003).

Although both groups showed statistically significant improvements, no significant between-group differences were observed at either point for weight and BMI.

### 3.3. Group Main Effects for Diabetes Knowledge

Table 5 presents the pre- to postprogram change in outcomes with significant group main effects. A significant main effect of group was observed for diabetes knowledge (*p* = 0.003), indicating that participants in the online cohorts consistently reported higher knowledge scores than those in the in-person cohorts. Pairwise comparisons further supported this finding, revealing significant between-arm differences at both preprogram (*p* = 0.025) and postprogram (*p* = 0.005) assessments.

Although no significant group × time interactions were detected, both groups demonstrated meaningful improvement in diabetes knowledge. Online participants improved from M = 78.0 to M = 87.0, while in-person participants showed an improvement from M = 74.4 to M = 83.1, representing gains of +9.0 and +8.7 points, respectively. Despite the similar magnitude of improvement, the online group maintained consistently higher scores at both time points.

While no other significant group × time interactions or group main effects were observed, Table 6 highlights the improvements in outcomes pre- to postprogram across both groups.

### 3.4. Clinical Outcomes

HbA1c: Online participants decreased their self-reported HbA1c by an average of −0.5%, from 7.5% to 7.0%. In-person participants decreased their HbA1c by an average of −0.5%, from 7.5% to 7.0%.

Self-efficacy: Diabetes management self-efficacy scores of online participants changed significantly from 7.1 points to 8.3 points and from 7.3 points to 8.1 points for in-person participants.

Self-care activities: In online participants, the number of days “following a healthy eating plan” increased from 4.2 to 5.1 days (+0.9 days) from pre- to postparticipation, and “eating fruits and vegetables/reducing consumption of red meat and full-fat dairy products” increased from 3.8 to 4.7 days (+0.9 days) from pre- to postparticipation. The self-care activity of “exercising” increased from 2.6 to 3.7 days (+1.1 days) from pre- to postparticipation in the online group. Additionally, “testing blood glucose levels” increased from 4.1 to 5.1 days (+1.0 days) from pre- to postparticipation for online participants, while “checking feet” increased from 3.2 to 4.5 days (+1.3 days) from pre- to postparticipation.

Comparatively, for participants attending in-person control programs, there was a significant increase from 4.0 to 4.9 days (+0.9 days) from pre- to postparticipation in “following a healthy eating plan.” Eating fruits and vegetables/reducing red meat and full-fat dairy increased from 3.9 days preparticipation to 4.7 days postparticipation (+0.8 days). Exercising increased from 2.6 to 3.6 days (+1.0 days) from pre- to postparticipation. There was also an increase from 4.4 days preparticipation to 5.2 days postparticipation (+0.8 days) in the number of days “testing their blood glucose levels,” and in “checking feet” from 3.8 days pre- to 4.7 days postparticipation (+0.9 days).

## 4. Discussion

Findings from pre- to post-HED program participation in both online and in-person programs showed high retention rates with positive changes across multiple domains, including biometric outcomes, self-efficacy, diabetes knowledge, and self-management behaviors. Significant improvements were observed in weight, BMI, and diabetes knowledge across both delivery formats. Although participants attending online programs and in-person programs improved clinical outcomes (like HbA1c), self-efficacy, and the five self-care behaviors, the differences between the groups were not statistically significant. However, diabetes knowledge scores were consistently higher in the online group compared to the control in-person group. These findings suggest that online HED programs are, at least, as effective in supporting knowledge acquisition, a key behavioral outcome, as traditional in-person delivery, reinforcing the viability of remote formats for diabetes education and management.

Our study found significant improvements in weight, BMI, and diabetes knowledge, as well as positive improvements in all other measures of program effectiveness in both online and in-person programs. These findings are consistent with prior research demonstrating the effectiveness of online-delivered diabetes education programs. Molavynejad et al. [11] concluded in a systematic review that online diabetes education is generally noninferior and, in some cases, superior to face-to-face education in improving outcomes such as glycemic control, knowledge acquisition, and self-care behaviors. Similarly, Molavynejad et al. [12] found that asynchronous, video-based telecare education was equally effective as in-person instruction in enhancing dietary compliance among adults with Type 2 diabetes. Broader evidence from other chronic disease contexts supports these findings. Rush et al. [29] demonstrated that telehealth-delivered educational interventions were efficacious across a range of chronic conditions, often leading to improvements in disease knowledge, self-management skills, quality of life, and clinical indicators. Additionally, Roberts et al. [30] in an umbrella review of healthcare delivery innovations reported that telehealth and remote models of care often yield outcomes comparable to or improved, such as patient knowledge and quality of life, compared to the traditional models, especially when tailored to patient needs. Collectively, these studies reinforce our findings that online HED program participants experienced comparable or greater gains in diabetes knowledge, weight, and BMI compared to in-person participants, supporting the viability of remote delivery models.

However, when comparing demographics of online and in-person programs, our study found differences in age, race, education level, and income level between participants who self-selected the online delivery method and those who selected in-person programs. Comparisons between adjusted and unadjusted models revealed that some outcome measures were more sensitive to delivery format (i.e., online vs. in-person) only after adjusting for covariates, underscoring the importance of accounting for demographic variation when interpreting program effects. Specifically, Recee et al. [31] found significant associations between income and diabetes outcomes, including HbA1c and cholesterol testing, foot exams by a physician, treatment for diabetes, and attending diabetes education classes. Similarly, González-Zacarias et al. [32] reported that income influences access to nutritious foods and the availability of healthcare supplies and services. In our study, participants in the in-person programs had lower income levels than those in the online program.

Findings from our study support suggestions by Roberts et al. [30] who emphasized that patient characteristics may be critical in determining suitability for online health program delivery methods. Allowing participants to self-select their preferred delivery format may enable them to tailor their learning experiences to their needs, schedules, and preferences, potentially increasing engagement and retention in DSMS programs. For example, younger individuals or those with irregular work schedules may find online programs more accessible and convenient, while older adults may prefer the socialization and tactile learning experiences that are associated with in-person formats. In both instances, our study found very high retention rates and comparable effectiveness across diabetes self-care outcomes for both delivery formats. Online DSMES programs offer the advantage of greater scalability, requiring fewer facilitators and reducing travel and time costs by extending reach across geographic regions. In contrast, in-person DSMES programs may have greater appeal for Black/African American participants and those with lower income and education levels. While our study did not assess participants' reasons for choosing a specific delivery format, further research on delivery method preferences could inform targeted outreach and promotional strategies to enhance engagement among diverse participant populations.

## 5. Strengths and Limitations

The strengths of this study include the large sample size of participants enrolled in an ADA practice-tested diabetes self-management support program with a community-clinical delivery model. The findings from this study suggest that online and in-person HED programs improve diabetes self-management outcomes regardless of demographic variables. Importantly, because delivery methods reached participants with different sociodemographic characteristics, this study provides insight into delivery modalities that appeal to different groups based on participants' characteristics such as age, ethnicity, education, and income levels.

As is common with community programs, there are some limitations to this study. Participants were included with a diagnosis of diabetes (or related risk), but the time since diagnosis and the degree of glycemic control at the beginning of the program were not determined. These are confounding variables that must be considered in the interpretation of the results. Most significantly, the program's delivery method was not randomized, as participants self-selected into either online or in-person format. As a result, our findings should be interpreted with the understanding that individuals who get to choose their preferred delivery method may be more motivated or suited to engage with HED compared to those who would have been assigned. Additionally, this study included only participants who completed the program and had completed pre- and postprogram data for the relevant outcomes. While this approach ensured consistency in measuring change, it may limit the generalizability of findings to all program enrollees, including those who did not complete the intervention or assessments. The quasiexperimental design with nonrandomized groups required adjustment for demographic covariates to reduce potential confounding. However, covariate adjustment does not fully eliminate the possibility of unmeasured or residual confounding, and group differences may still have influenced the results. These findings should therefore be interpreted with this limitation in mind.

Finally, the study was conducted in a single state in the southeastern region of the United States, which may limit generalizability to other regions. A multistate expansion of the HED program delivered online and in-person is currently in progress. Additionally, the Spanish-HED program was only available online during the study period, which likely contributed to the higher proportion of Hispanic participants in the online group and may have influenced comparisons between delivery formats.

## 6. Conclusions

Three primary conclusions can be drawn from this study. First, both online and in-person HED programs showed significant improvements in within-arm outcomes. Individuals in both delivery formats demonstrated improvements in clinical outcomes (e.g., weight, BMI, and HbA1c), diabetes knowledge, self-efficacy, and self-care behaviors. This suggests that the HED DSMS program can be delivered in-person or online with similar outcome results. Secondly, participants who self-selected into online vs. in-person formats differed significantly in age, race, education, and income. Some outcome differences only emerged after adjusting for these covariates, underscoring the importance of accounting for demographic variation when evaluating program effects. Given the comparable effects on both formats, offering both delivery methods maximizes access to DSMS programs for a range of ages, races and ethnicities, incomes, and education levels. And thirdly, the implementation of the HED program model, utilizing a community-clinical linkage in which extension agents serve as health paraprofessionals in collaboration with CDCES professionals, appears to support positive health outcomes among participants in both online and in-person HED DSMS programs.

## Figures and Tables

**Table 1:** Retention rates.

	**Online group (%)**	**In-person group (%)**	**p** ** value**
Raw retention rate, mean (Std.)	74.4 (18.5)	76.7 (21.4) and 79.60 (17.45)	0.480
Attending 2+ education sessions retention rate, mean (Std.)	78.7 (17.5)	83.7 (15.7)	0.069

*Note:* Raw retention rate is the total number of all individuals who graduated from HED divided by the total number of all individuals who completed enrollment. Attending 2+ education sessions retention rate is the total number of individuals who attended two or more HED educational sessions divided by the total number of all individuals who completed enrollment.

**Table 2:** Demographics for participants in online and in-person cohorts.

**Demographic variables**	**Intervention online**	**Standard in-person**	**p** ** value**
	**N** = 613	**N** = 405	**< 0.001**⁣^∗^
Age, mean (Std.)	62.4 (±12.1)	69.3 (±11.8)	
Biological sex, *N* (%)			0.389
Male	174 (28.4)	105 (25.9)	
Female	439 (71.6)	300 (74.1)	
Race, *N* (%)			**< 0.001**⁣^∗^
African American/Black	145 (23.7)	205 (50.6)	
White	397 (64.8)	185 (45.7)	
Other	64 (10.4)	11 (2.7)	
Prefer not to answer	7 (1.1)	4 (1.0)	
Ethnicity, *N* (%)			**< 0.001**⁣^∗^
Non-Latino/Hispanic	524 (85.5)	384 (94.8)	
Latino/Hispanic	67 (10.9)	9 (2.2)	
Other	13 (2.1)	2 (0.5)	
Prefer not to answer	9 (1.5)	10 (2.5)	
Education level, *N* (%)			**< 0.001**⁣^∗^
Less than high school	27 (4.4)	8 (2.0)	
Some high school	10 (1.6)	27 (6.7)	
High school diploma/GED	83 (13.5)	103 (25.4)	
Some college	117 (19.1)	92 (22.7)	
Technical/associate degree	89 (14.5)	64 (15.8)	
Bachelor's degree	134 (21.9)	56 (13.8)	
Some postgraduate education	153 (25.0)	55 (13.6)	
Annual income, *N* (%)			**< 0.001**⁣^∗^
Less than $15,000	76 (18.8)	44 (7.2)	
$15,000–$25,000	60 (14.8)	51 (8.3)	
$25,000–$49,999	74 (18.3)	118 (19.2)	
$50,000–$74,999	38 (9.4)	79 (12.9)	
$75,000–$99,999	15 (3.7)	51 (8.3)	
Greater than $100,000	14 (3.5)	51 (8.3)	
Prefer not to answer	128 (31.6)	219 (35.7)	
Diabetes type, *N* (%)			0.066
Type 1	19 (3.1)	15 (3.7)	
Type 2	571 (93.1)	366 (90.4)	
Do not know	13 (2.1)	20 (4.9)	
Not diagnosed presently	10 (1.6)	4 (1.0)	

*Note: *Bolded *p* values indicate statistically significant group differences.

⁣^∗^*p* values represent the results of independent samples *t*-tests for continuous variables and chi-square tests of independence for categorical variables.

**Table 3:** Results of adjusted MANCOVA and ANCOVA analyses for group × time interactions and group main effects.

	**G** **r** **o** **u** **p** × **time interaction** **p value (effect size)**	**Group main effect** **p** ** value (effect size)**
MANCOVA		
Clinical outcomes	**0.007 (0.010)**	0.630 (0.001)
Weight	**0.002 (0.010)**	0.259 (0.001)
BMI	**0.002 (0.010)**	0.331 (0.001)
Psychosocial outcomes	0.074 (0.005)	**0.009 (0.009)**
Diabetes knowledge	0.944 (0.000)	**0.003 (0.009)**
Diabetes self-efficacy	0.024 (0.005)	0.392 (0.001)
Self-care behaviors	0.687 (0.003)	0.615 (0.004)
ANCOVA		
Clinical outcomes		
HbA1c	0.482 (0.001)	0.984 (0.000)

*Note:* Bolded *p* values indicate statistically significant effects (*p* < 0.05). Effect sizes are partial eta squared.

**Table 4:** Pre- to postprogram change for outcomes with significant group × time interactions.

**Group**	**Intervention online**				**Standard in-person**				**Effect size**
**Time**	**Preprogram**	**Postprogram**	**Change pre- to post-HED**	**p** ** value**	**Preprogram**	**Postprogram**	**Change pre- to post-HED**	**p** ** value**	
Clinical outcomes, mean (Std.)									0.010
Weight	211.6 (±53.5)	206.9 (±53.0)	−4.7	**< 0.001**	208.4 (±48.6)	206.3 (±48.4)	−2.1	**0.002**	
BMI	34.4 (±7.6)	33.6 (±8.2)	−0.8	**< 0.001**	33.9 (±7.6)	33.6 (±7.6)	−0.3	**0.003**	

*Note:* Bolded values indicate statistically significant within-group changes (*p* < 0.05). Effect size reflects interaction term (partial eta squared).

**Table 5:** Pre- to postprogram change for outcomes with significant group main effects.

**Group**	**Preprogram**				**Postprogram**				**Effect size**
**Time**	**Online**	**In-person**	**Difference between groups**	**p** ** value**	**Online**	**In-person**	**Difference between groups**	**p** ** value**	
Psychosocial outcomes, mean (Std.)									0.009
Diabetes knowledge	78.0 (±13.8)⁣^∗^	74.4 (±14.6)	−3.6	0.025	87.0 (±9.6)⁣^∗∗^	83.1 (±12.6)	−3.9	0.005	

*Note:* Group main effect was significant for diabetes knowledge (*p* = 0.004), indicating that the online group had consistently higher knowledge scores across time points.

⁣^∗^Significant difference between groups at preprogram (*p* < 0.05).

⁣^∗∗^Significant difference between groups at postprogram (*p* < 0.05).

**Table 6:** Pre- to postprogram change in outcomes without significant group × time or group main effects.

**Group**	**Intervention online program**			**Standard in-person program**		
**Time**	**Preprogram**	**Postprogram**	**Change pre- to post-HED**	**Preprogram**	**Postprogram**	**Change pre- to post-HED**
Clinical outcomes, mean (Std.)						
HbA1c	7.5 (±1.6)	7.0 (±1.2)	−0.5	7.5 (±1.6)	7.0 (±1.1)	−0.6
Psychosocial outcomes, mean (Std.)						
Diabetes self-efficacy	7.1 (±1.7)	8.3 (±1.1)	1.2	7.3 (±1.8)	8.1 (±1.4)	0.9
Self-care behaviors, mean (Std.)						
General diet	4.2 (±2.3)	5.1 (±1.3)	0.9	4.0 (±2.3)	4.9 (±1.5)	0.9
Specific diet	3.8 (±1.8)	4.7 (±1.5)	0.9	3.9 (±1.8)	4.7 (±1.5)	0.8
Exercise	2.6 (±2.2)	3.7 (±2.0)	1.1	2.6 (±2.2)	3.6 (±2.0)	1.0
Blood glucose monitoring	4.1 (±2.8)	5.1 (±2.4)	0.9	4.5 (±2.7)	5.2 (±2.3)	0.7
Footcare	3.2 (±2.3)	4.5 (±2.1)	1.3	3.8 (±2.4)	4.6 (±2.2)	0.8

## Data Availability

The datasets used and/or analyzed during the current study are available from the corresponding author upon reasonable request.
